# Calretinin and Neuropeptide Y interneurons are differentially altered in the motor cortex of the SOD1^**G93A**^ mouse model of ALS

**DOI:** 10.1038/srep44461

**Published:** 2017-03-15

**Authors:** Rosemary M. Clark, Catherine A. Blizzard, Kaylene M. Young, Anna E. King, Tracey C. Dickson

**Affiliations:** 1 Menzies Institute for Medical Research, University of Tasmania, Hobart, 7000, Australia; 2 Wicking Dementia Research & Education Centre2, University of Tasmania, Hobart, 7000, Australia

## Abstract

Increasing evidence indicates an excitatory/inhibitory imbalance may have a critical role in the pathogenesis of amyotrophic lateral sclerosis (ALS). Impaired inhibitory circuitry is consistently reported in the motor cortex of both familial and sporadic patients, closely associated with cortical hyperexcitability and ALS onset. Inhibitory network dysfunction is presumably mediated by intra-cortical inhibitory interneurons, however, the exact cell types responsible are yet to be identified. In this study we demonstrate dynamic changes in the number of calretinin- (CR) and neuropeptide Y-expressing (NPY) interneurons in the motor cortex of the familial hSOD1^G93A^ ALS mouse model, suggesting their potential involvement in motor neuron circuitry defects. We show that the density of NPY-populations is significantly decreased by ~17% at symptom onset (8 weeks), and by end-stage disease (20 weeks) is significantly increased by ~30%. Conversely, the density of CR-populations is progressively reduced during later symptomatic stages (~31%) to end-stage (~36%), while CR-expressing interneurons also show alteration of neurite branching patterns at symptom onset. We conclude that a differential capacity for interneurons exists in the ALS motor cortex, which may not be a static phenomenon, but involves early dynamic changes throughout disease, implicating specific inhibitory circuitry.

Interneurons play a crucial role in balancing neuronal activity in the brain^1^. In the devastating neurodegenerative disease amyotrophic lateral sclerosis (ALS), the loss of inhibitory interneuronal activity has been associated with the development of cortical hyperexcitability, linked to the onset of motor neuron degeneration that characterises the disease (for reviewed in^2^,^3^,^4^). Present in both sporadic and familial forms of ALS^5^,^6^,^7^,^8^,^9^,^10^, inhibitory dysfunction manifests in the motor cortex as reduced short-interval intracortical inhibition (SICI), which in combination with increased glutamate activity, is thought to cause cortical hyperexcitability^11^,^12^. This pathophysiological phenomenon is identified in the motor cortex of patients prior to lower motor neuron dysfunction^13^; suggesting early changes in the balance between excitation and inhibition in the cortex may initiate, or in the very least, exacerbate, ALS pathology.

As a determinant of disease progression, clinical studies show that sporadic patients with greater intracortical inhibitory dysfunction have a more rapid clinical decline and shorter disease duration^14^. Similarly, familial SOD1 mutation carriers with preserved intracortical inhibitory circuitry have a slower disease progression^15^, surviving an average of 13 years compared to 3 in sporadic patients^16^,^17^. This indicates that for both sporadic and familial ALS, the preservation, or restoration, of inhibition could be a promising neuro-protective strategy. However, little is known about the underlying architecture that may be initiating inhibitory dysfunction in the cortex, particularly the cellular components involved.

Within the cortex, inhibition and the regulation of excitability is provided by a group of heterogeneous cells, the interneurons^18^,^19^, which have the potential to underlie inhibitory dysfunction in the ALS cortex. It is known that essential components of the inhibitory system are altered in ALS. The main inhibitory neurotransmitter, γ-aminobutyric acid (GABA) is reduced in the motor cortex of ALS patients^20^, mRNA levels of the prominent GABA_A_ receptor subunit alpha-1 are reduced^21^ and pet scans reveal reduced binding of the GABA receptor ligand, flumazenil, in the motor cortex of sporadic ALS patients^22^. Furthermore, the reduced SICI observed in patients is thought to reflect changes in the function of inhibitory GABA-secreting cortical interneurons, as well as the circuits they contribute to^11^,^12^. The pathological consequence of this is demonstrated by the concurrent loss of GABAergic activity in the motor cortex, alongside elevated levels of glutamate^23^, which is proposed to cause motor neuron degeneration through the trans-synaptic anterograde propagation of glutamate toxicity^13^,^24^. However, despite increasing evidence that interneurons are likely of central importance in ALS pathophysiology, it is poorly understood which types of interneurons are involved, and therefore, how specific interneuronal networks are implicated in disease.

To address these questions, this study aimed to determine the extent and timing of interneuron pathology in the cortex of the SOD1^G93A^ mouse model of ALS^25^. This mouse model develops prominent motor symptoms, has a well documented history of disease progression^26^, and was recently used to identify early cortical motor dysfunction^27^, including increased excitability of layer V pyramidal neurons in the motor cortex^28^, which preceded lower motor neuron symptoms^29^ and degeneration of cortico-spinal motor neuron pathways^30^. However, the potential for an underlying interneuronal phenotype has not yet been fully explored in this ALS model. Therefore, using immunohistochemistry and cell tracing software, we investigated if there was a region-dependent vulnerability of specific interneuron populations in the cortex of the SOD1^G93A^ mouse. We report a substantial loss of CR-expressing interneurons, specifically in the SOD1^G93A^ motor cortex, with differential vulnerability of NPY-expressing interneurons at late-disease stages, potentially reflecting compensatory mechanisms. In addition, the early symptomatic involvement of both CR- and NPY-interneurons is demonstrated, supporting an early and progressive contribution of inhibitory circuits throughout disease. Collectively, this data suggests NPY- and CR-interneurons are involved in a motor-specific inhibitory phenotype from early stages in disease – thereby demonstrating the potential for an underlying inhibitory contribution in the SOD1 mouse model of ALS.

## Results

### A subtype-specific alteration of interneurons is evident after symptom onset in the SOD1 motor cortex

**Figure 1:**
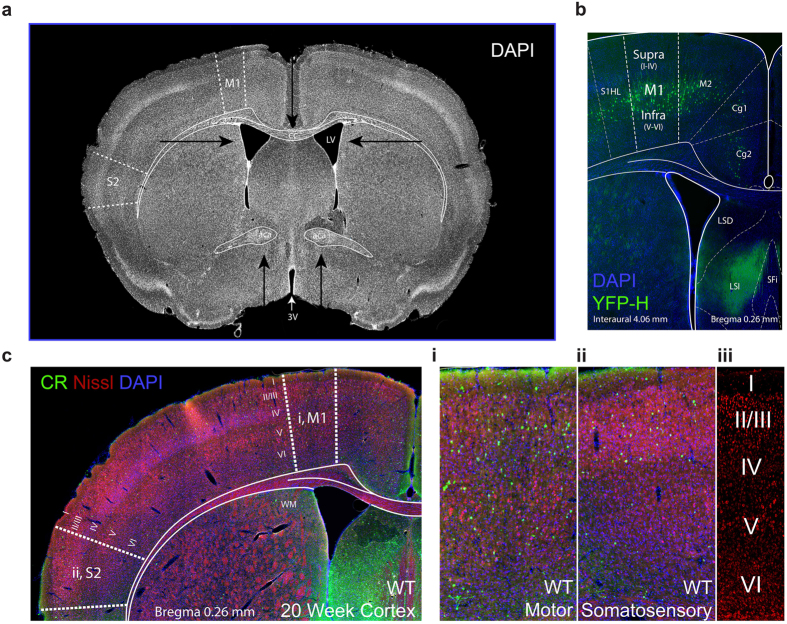
Representative images of sections used for quantitative analysis in WT and SOD1^G93A^ mice. Coronal sections, each 40 μm thick, from bregma 1.18 to −0.58 were used for quantitative analysis at four stages of disease progression in each cohort of mice. (**a**) An example section used in the study (Interaural 4.06 mm, Bregma 0.26 mm) with primary motor (M1) and secondary somatosensory (S2) regions denoted by dotted lines. Arrows indicate anatomical landmarks used to identify regions of interest, as visualised by DAPI staining: namely, the namely the third (3 V) and lateral ventricles (LV), shape and appearance of the corpus callosum (cc) and anterior commissure (aca). (**b**) The motor cortex imaging site as validated in the Thy1-eYFP-H mouse, which has particularly prominent yellow fluorescent protein expression in large layer V corticospinal neurons (green) within the motor cortex^57^,^80^. (**c**) A representative image of a calretinin (green), Nissl (red), DAPI (blue) cortical coronal section used for analyses. In each section, the motor (i) and somatosensory (ii) regions of interest (boxed areas enlarged to the right) were used for quantitative analyses, with cortical layers I–VI visualised by Nissl staining (iii).

**Figure 2:**
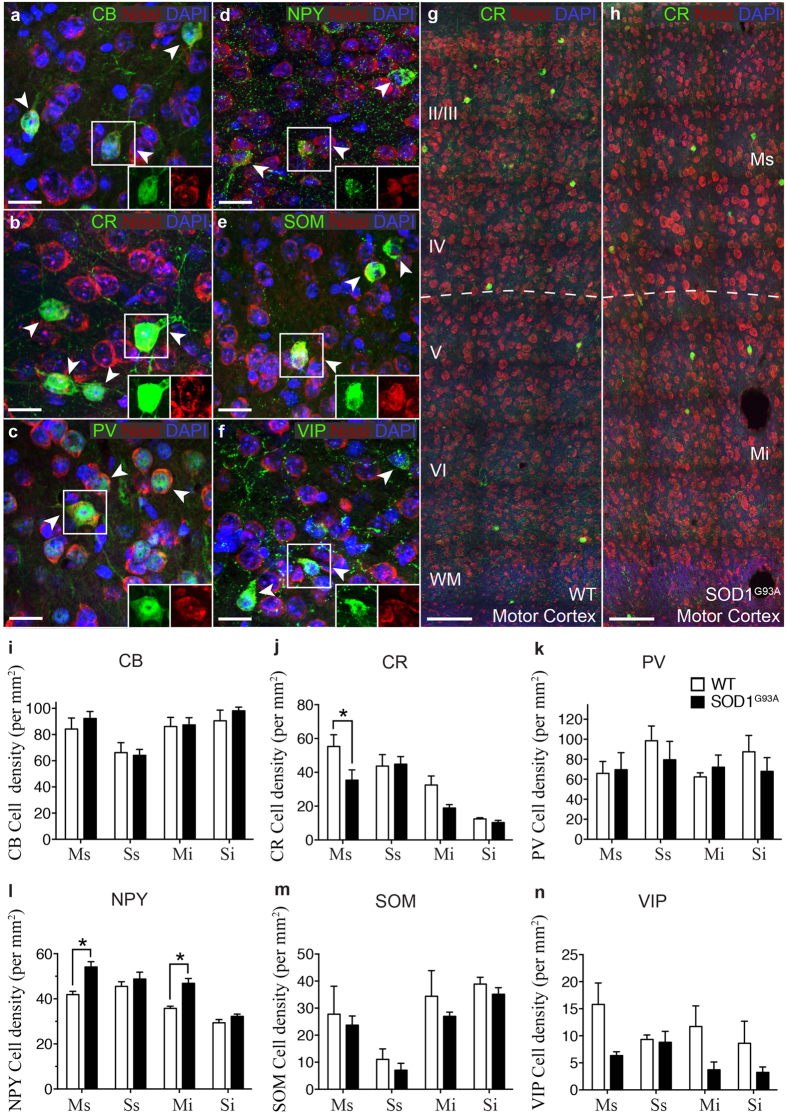
Calretinin and Neuropeptide Y interneuron subtypes are differentially altered in specific lamina of the SOD1^G93A^ motor cortex. (**a–f**) Calcium binding proteins and neuropeptides (green) were used to visualise specific interneuron populations in the cortex, showing labelling patterns of calbindin (CB; **a**) calretinin (CR; **b**) parvalbumin (PV; **c**) and neuropeptide Y (NPY; **d**) somatostatin (SOM; **e**) and vasoactive intestinal peptide (VIP; **f**) populations in 20 week WT cortex stained with DAPI (blue) and Nissl (red). The boxed areas (**a–f**) in the high magnification images show co-localisation of interneuron labels with Nissl stain. (**g,h**) At 20 weeks, analysis of motor cortex, reveals the normal distribution of CR-interneurons in WT motor cortex (**g**) but a striking reduction in particular in layers I-IV of SOD1^G93A^ motor cortex. (**h**) Analysis of immunopositive neurons within the SOD1^G93A^ motor (M) and somatosensory (S) cortex showed that the density of calretinin-expressing interneurons was significantly decreased specifically within the supragranular (Ms, layers I-IV) lamina of the motor cortex (**j**) and the density of Neuropeptide Y-expressing interneurons was significantly increased in both the supragranular (Ms, layers I-IV) and infragranular lamina (Mi, layers V-VI) of the motor cortex. (**l**) No other interneuron populations were significantly altered in either motor or somatosensory cortex. (**i**,**k**,**m**,**n**) Values in graphs represent means ± SEM. *P < 0.05, two-way ANOVA, Bonferonni’s multiple-comparison test with n = 6 mice per group. Scale bar in (**a–f**) 20 μm, (**g–h**) 100 μm.

Within the cortex, inhibitory microcircuits are comprised of a wide variety of interneuron populations that target specific neuronal domains to facilitate the fine-tuning of cortical neuronal activity. These cell types are arranged in well-ordered wiring patterns that maintain the complex functions of cortical regions by their unique placement, connections and firing properties^1^. Changes in specific interneuron populations are therefore likely to affect synaptic transmission in the motor cortex and compromise the regulation of network excitability, including motor output from layer V corticomotoneurons. To determine if specific interneuron populations were altered in the SOD1 cortex, we used immunohistochemistry to assess the potential changes in the numbers of interneuron populations in the motor and somatosensory cortex of late-symptomatic (20 week) SOD1 mice, and in age and litter-matched wild type (WT) controls. We quantified interneuron density (cells per mm^2^) in both the supragranular and infragranular lamina of motor (Ms, Mi) and somatosensory cortices (Ss, Si) to determine if cell position in cortical regions influenced pathology (Fig. 1a–c). GABAergic interneuron subtypes were differentiated according to the selective expression of calcium binding proteins [calbindin (CB), calretinin (CR), parvalbumin (PV)] and neuropeptides [neuropeptide Y (NPY), somatostatin (SOM), vasoactive intestinal peptide (VIP)]^19^ (Fig. 2a–f). We found that of the interneuron populations expressing calcium-binding proteins, the density of CR-expressing neurons was significantly decreased in the supragranular lamina of the motor cortex (layers I-IV) (Fig. 2g,h). In this region, CR-neurons were reduced by up to 37% of WT controls (55 ± 6 p/mm^2^ WT Ms, 35 ± 6 p/mm^2^ SOD1^G93A^ Ms) (*P* < 0.05, two-way ANOVA, Bonferroni post-hoc) (Fig. 2j), while the density of this population remained unaltered in the infragranular motor cortex (32 ± 5 p/mm^2^ WT Mi, 19 ± 1.9 p/mm^2^ SOD1^G93A^ Mi), and unaltered in both lamina of the somatosensory cortex (43 ± 6 p/mm^2^ WT Ss, 44 ± 4 p/mm^2^ SOD1^G93A^ Ss; 12 ± 1 p/mm^2^ WT Si, 10 ± 1 p/mm^2^ SOD1^G93A^ Si). No significant differences were detected in either of the other calcium binding populations, CB- or PV-expressing neurons in SOD1^G93A^ mice and WT controls (Fig. 2i,k). In direct contrast, the number of NPY-expressing neurons was significantly increased by 29% in the supragranular motor cortex (41 ± 1 p/mm^2^ WT Ms, 54 ± 2 p/mm^2^ SOD1^G93A^ Ms) and by 30% in the infragranular motor cortex of SOD1^G93A^ mice compared with WT (35 ± 1 p/mm^2^ WT Mi, 46 ± 2 p/mm^2^ SOD1^G93A^ Mi) (*P* < 0.05, two-way ANOVA, Bonferroni post-hoc) (Fig. 2l), an unexpected finding. In the somatosensory cortex NPY-neurons remained unchanged in both lamina (45 ± 2 p/mm^2^ WT Ss, 48 ± 3 p/mm^2^ SOD1^G93A^ Ss; 29 ± 1 p/mm^2^ WT Si, 32 ± 1 p/mm^2^ SOD1^G93A^ Si). Analysis of other neuropeptide expressing populations, SOM- and VIP-expressing neurons, identified no further differences in motor or somatosensory lamina compared to WT (Fig. 2m,n). These investigations demonstrate that at end-stage in the SOD1^G93A^ cortex, at a time of established cortical vulnerability in this ALS model^30^, distinct regions of the motor cortex undergo selective alteration involving the differential vulnerability of neurons expressing CR and NPY.

### Contrasting and progressive alterations of NPY and CR populations throughout the SOD1^G93A^ time course

**Figure 3:**
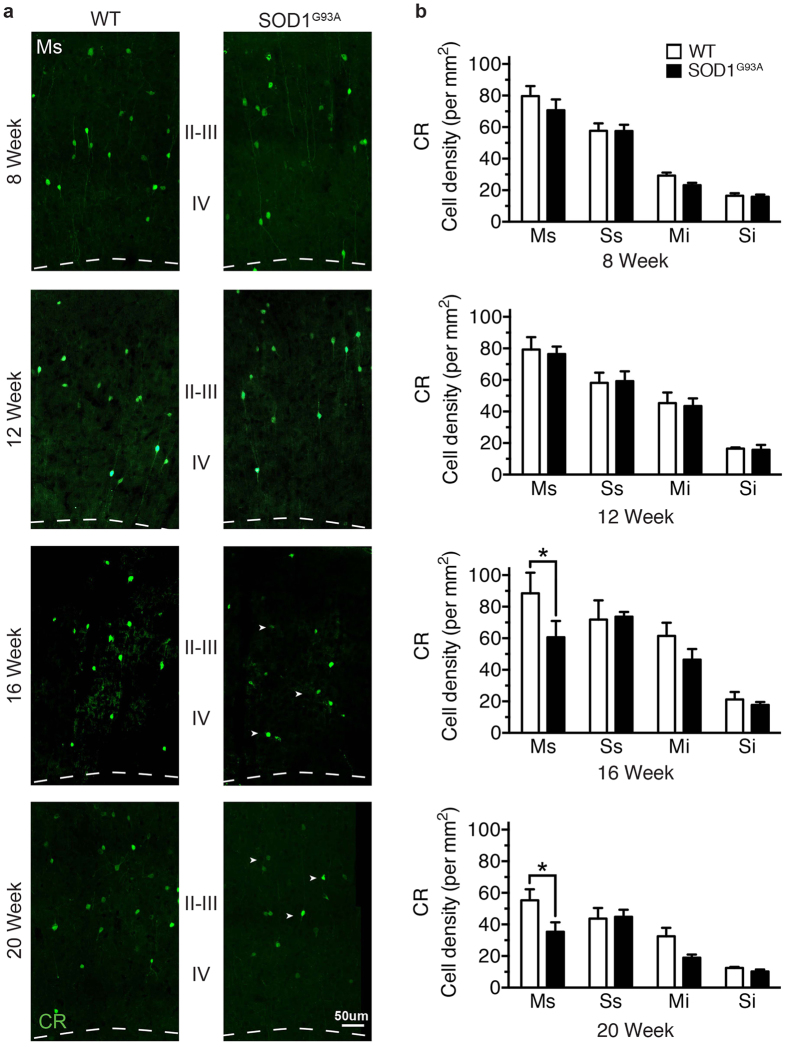
Calretinin-expressing interneurons are progressively lost during the symptomatic phase in the SOD1^G93A^ motor cortex. (**a**) CR-expressing neurons were labelled throughout the SOD1^G93A^ disease course, showing neurons were present at comparable levels in SOD1^G93A^ and WT mice at 8 weeks (early symptom onset) and 12 weeks in motor (M) and somatosensory cortex (S). (**b**) Analysis of 16 week symptomatic SOD1^G93A^ mice showed that CR neurons were significantly decreased in the supragranular lamina of motor cortex (Ms, layers I-IV) compared to WT mice. CR-neurons were progressively reduced in the supragranular lamina of motor cortex (Ms, layers I-IV) in 20 week end-stage SOD1^G93A^ mice (arrow heads in **a**). Values in graphs represent means ± SEM. *P < 0.05, two-way ANOVA, Bonferonni’s multiple-comparison test with n = 6 mice per group. Scale bar in (**a**) 50 μm.

The early alteration of interneuron populations in the motor cortex could initiate a cascade of events resulting in an inability to maintain excitability in the cortex. In the TDP-43 model of ALS, SOM-expressing interneurons has been shown to initiate hyperexcitability in the motor cortex at an early disease stage^31^. We therefore examined the density of CR- and NPY-expressing populations at earlier stages in the SOD1^G93A^ disease course: 8 weeks (from the earliest signs of symptoms in this model), 12 weeks and 16 weeks^26^ (Fig. 3a). The mean density of CR-expressing neurons was decreased by 31% from (16 weeks) in the supragranular motor cortex of SOD1^G93A^ mice compared with WT (88 ± 13 p/mm^2^ WT Ms, 60 ± 8 p/mm^2^ SOD1^G93A^ Ms) (*P* < 0.05, two-way ANOVA, Bonferroni post-hoc) (Fig. 3b). This decrease was not significant in the infragranular lamina of the motor cortex (61 ± 8 p/mm^2^ WT Ms, 46 ± 6 p/mm^2^ SOD1^G93A^ Ms), and was not present in the somatosensory cortex (71 ± 12 p/mm^2^ WT Ss, 48 ± 11 p/mm^2^ SOD1^G93A^ Ss; 21 ± 9 p/mm^2^ WT Si, 17 ± 2 p/mm^2^ SOD1^G93A^ Si). The density of CR-expressing neurons remained unchanged relative to controls at earlier time points in the SOD1^G93A^ cortex at 8 and 12 weeks. This suggests either a loss of CR-expressing neurons, or a potential reduction in the expression levels of CR in this distinct interneuron population, occurs during the later symptomatic phase in this model and is restricted to the upper layers of the motor cortex.

**Figure 4:**
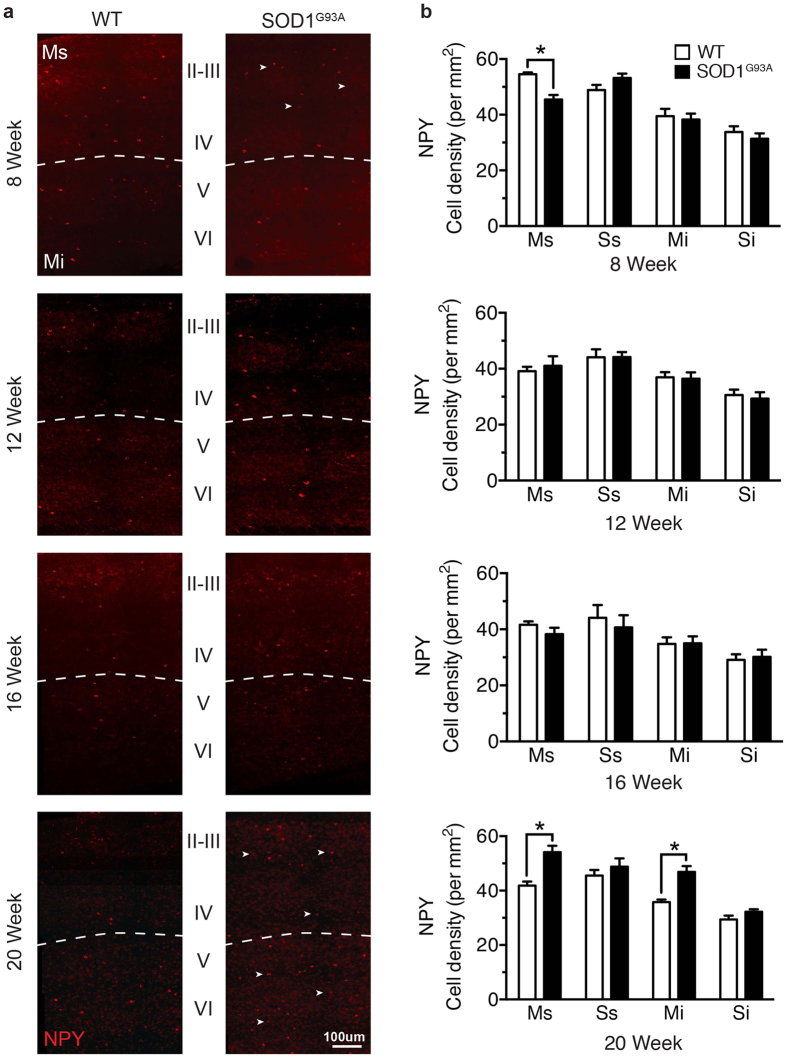
Neuropeptide Y populations are differentially altered from early symptom onset in the SOD1^G93A^ motor cortex. (**a**) NPY-expressing neurons were labelled at different stages in the SOD1^G93A^ disease course: 8 weeks (early symptom onset), 12 weeks,16 weeks and 20 weeks (end-stage). (**b**) The mean density of NPY-expressing neurons was significantly decreased in the supragranular lamina of the motor cortex (Ms, layers I-IV) in early symptomatic (8 week) SOD1^G93A^ mice compared to WT (arrow heads in **a**). NPY-expressing neurons were present at comparable levels in SOD1^G93A^ and WT mice at 12 weeks and 16 weeks, with a late increase in cell density detected throughout the motor cortex at 20 weeks in end-stage SOD1^G93A^ mice (arrow heads in **a**). The somatosensory cortex showed no change in NPY-expressing neurons at any stage of disease in supragranular (Ss, layers I-IV) or infragranular lamina (Si, layers V-VI) in SOD1^G93A^ and WT mice. Values in graphs represent means ± SEM. *P < 0.05, two-way ANOVA, Bonferonni’s multiple-comparison test with n = 6 mice per group. Scale bar in (**a**) 100 μm.

Interestingly, in contrast to the end-stage increase in NPY-expressing neurons in the SOD1^G93A^ motor cortex (Fig. 2d), from symptom onset (8 weeks) NPY-neurons were significantly decreased by 17%, but only in the supragranular lamina of the SOD1^G93A^ motor cortex (55 ± 1 p/mm^2^ WT Ms, 45 ± 2 p/mm^2^ SOD1^G93A^ Ms) (*P* < 0.05, two-way ANOVA, Bonferroni post-hoc) (Fig. 4a,b). At the same time point NPY-neurons remained unaltered in the infragranular lamina of the motor cortex (39.5 ± 3 p/mm^2^ WT Ms, 38 ± 2 p/mm^2^ SOD1^G93A^ Ms), and in other somatosensory regions (49 ± 2 p/mm^2^ WT Ss, 53 ± 2 p/mm^2^ SOD1^G93A^ Ss; 33 ± 2 p/mm^2^ WT Si, 31 ± 2 p/mm^2^ SOD1^G93A^ Si). Notably, NPY-expressing neurons were also unaltered in all cortical regions investigated at 12 and 16 weeks. These data suggest that changes to interneurons can occur early in the SOD1^G93A^ motor cortex, is progressive with CR-expressing neurons, and may alter over the disease course in relation to NPY-expressing neurons.

### Early and progressive alteration of CR-labelled neurites in the supragranular SOD1^G93A^ motor cortex

**Figure 5:**
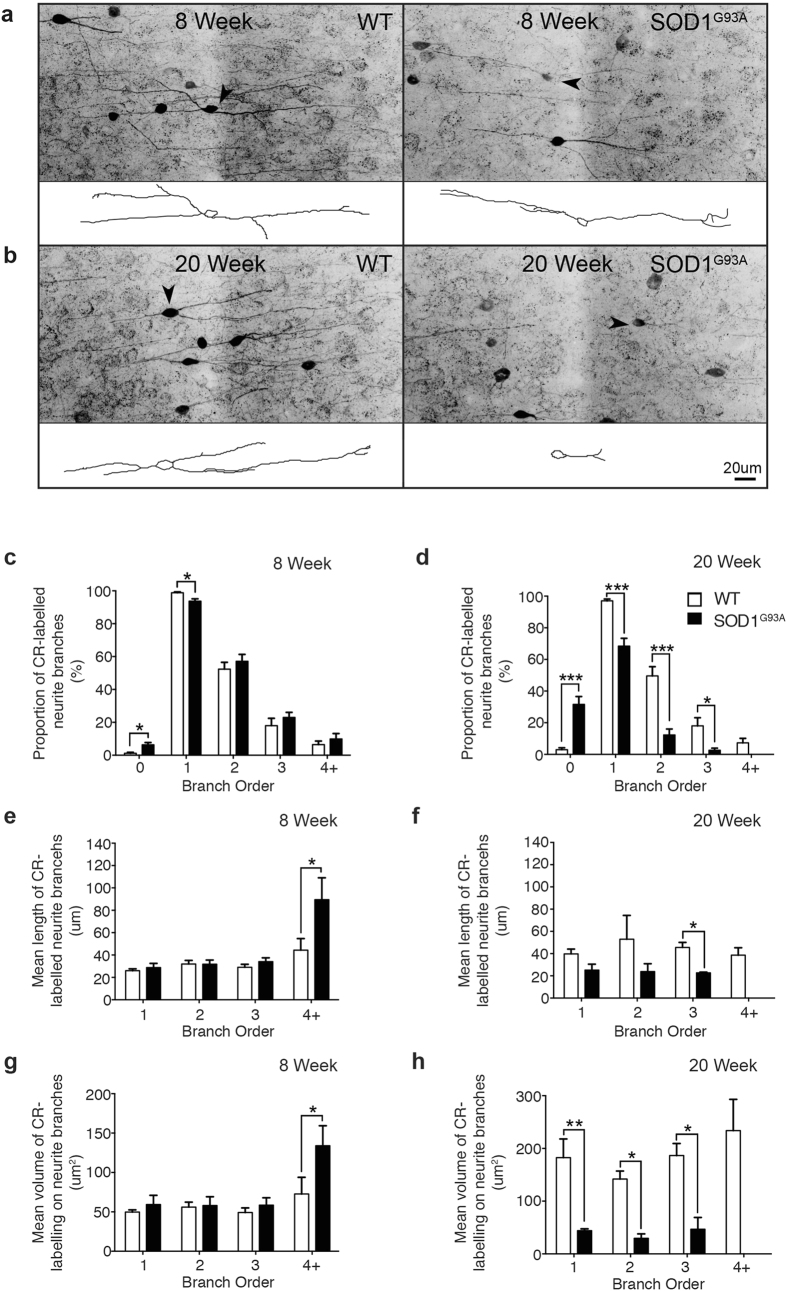
Calretinin-expressing neurites undergo progressive alterations in branching complexity from symptom onset in the supragranular SOD1^G93A^ motor cortex. (**a–d**) CR-labelled tissue sections were imaged and immunopositive neurons in the supragranular lamina assessed using cell tracing software to analyse neurite morphology (arrowheads, insets) at 8 weeks (**a**) and 20 weeks (**b**) in WT and *SOD1*^*G93A*^ tissue. The division of the neurite structure into primary, secondary, tertiary and quaternary order processes (as observed with CR-labelling) was used for all investigations. (**c**,**d**) A proportional analysis was conducted to determine the extent of CR-labelled processes remaining on neurons in the supragranular motor cortex at both time points, showing a significant increase in neurons with no, or fewer, CR-labelled processes visible in early symptomatic (8 week) SOD1^G93A^ mice and in 20 week end-stage SOD1^G93A^ mice compared to WT. (**e–h**) The mean branch length (**e,f**) and mean volume (**g,h**) of CR-labelled neurites were also assessed, demonstrating a pre-symptomatic increase in the length and volume of distal neurite processes in 8 week SOD1^G93A^ mice (**e**,**g**). Analysis of 20 week end-stage SOD1^G93A^ mice showed a significant reduction in the length and volume of CR-labelled processes compared to WT (**f**,**h**). Values in graphs represent means ± SEM. *P < 0.05, two-way ANOVA, Bonferonni’s multiple-comparison test with n = 6 mice per group. Scale bar in (**b**) 20 μm.

A characteristic hallmark of neuronal degeneration is the alteration of neurite structure, which can precede overt changes in cell number, while augmenting connectivity patterns and the innervation field of neuronal populations^32^,^33^,^34^,^35^,^36^. Due to the progressive nature of CR-cell alterations, we investigated whether CR-expressing neurons were abnormal in SOD1^G93A^ mice by examining branching patterns in 40um coronal sections at two contrasting disease stages: an early symptomatic time point (8 weeks), prior to loss of CR-expressing neurons demonstrated at 16 weeks, and an end-stage time point, when CR-neuron loss has been established for a prolonged period in the SOD1^G93A^ disease course. Immunolabeling of the supragranular lamina of the motor cortex revealed extensive differences between CR-labelled neurites from end-stage (20 week) SOD1^G93A^ mice and WT controls. CR-neurons in WT tissue had well-developed and extensive neurite trees, whereas the same neurons in SOD1^G93A^ tissue exhibited a substantial reduction in neurite labelling (Fig. 5b). Quantification confirmed this observation, revealing a significant reduction in the proportion of CR-positive neurons with primary (~70%), secondary (~25%) and tertiary (~14%) order processes in the SOD1^G93A^ cortex compared with WT (Fig. 5d). This was accompanied by a nine-fold increase in the proportion of SOD1^G93A^ CR-neurons with no neurites visible (Fig. 5d). Likewise, the average length of tertiary order processes (~48%) (Fig. 5f) and the average area of primary (~75%), secondary (~79%) and tertiary (~75%) order processes (Fig. 5h) was significantly decreased in the SOD1^G93A^ motor cortex compared to WT. Overall group comparisons revealed that the SOD1^G93A^ genotype significantly reduced the average length (~38%) and volume (~78%) of all CR-labelled neurites, independent of branch order (Fig. 5f,h). These results demonstrate that at end-stage the remaining population of CR-expressing neurons are irregular with reduced distribution of CR-immunoreactivity in their processes, which may translate into abnormal function in the motor cortex.

We next evaluated the CR-labelling patterns in the early symptomatic tissue (at 8 weeks) to establish if alterations were present prior to loss of CR-expressing neurons. Cell tracing revealed a similar, although more subtle, pattern of alterations to SOD1^G93A^ CR-neurons at this time point (Fig. 5a). There was a significant reduction in the proportion of CR-neurons with primary neurites (~5%) and a small increase in neurons with no processes visible (Fig. 5c). However, there was a trend towards a two-fold increase in the average length and area of neurites of CR-neurons in SOD1^G93A^ tissue, which was only significant in quaternary order processes (Fig. 5e,g). These results strongly suggest a potential involvement of CR-networks in relation to motor neuron circuitry defects previously observed in the motor cortex.

## Discussion

ALS is a system degeneration disorder characterised by the selective loss of both upper (corticospinal) and lower (spinal) motor neurons^37^. While lower motor neuron dysfunction and loss has been well characterised in animal models of ALS^25^,^29^,^38^,^39^, it is only in recent years that the potential contribution of cortical pathology has begun to be investigated^27^,^40^. Recent studies show that corticospinal motor neurons mirror some of the earliest signs of degeneration that occur in lower motor neurons^41^, including early electrophysiological changes, dendritic regression and cell loss^42^,^43^,^44^. Additionally, dysfunctional astrocytes, microglia and oligodendrocytes have been shown to contribute to disease progression and motor neuron degeneration in SOD1 mice^45^,^46^,^47^,^48^,^49^,^50^, increasing interest in the role of cortical neuronal and non-neuronal populations in these models^46^,^51^. Indeed, with the recent detection of early hyperexcitability in the motor cortex of patients, these and other studies support a cortical origin of disease^4^,^6^,^9^, initiated by cortical dysfunction, subsequently spreading to spinal motor neurons^6^,^52^. As such, there is increasing interest in the cortical components that may regulate motoneuronal circuitry, including the diverse cortical interneuron populations that underpin extrinsic regulation of excitability in the cortex^3^,^53^. Therefore, we investigated interneuron populations over a time course to establish potential involvement in the disease. The interneuron pathology reported here strongly support a sequence of inhibitory alteration, which is restricted to the motor cortex, includes specific interneuron populations in the SOD1 mouse model, and begins early in disease at a stage when, according to previous reports, corticospinal motor neurons are altered^30^.

Changes in CR- and NPY-expressing interneuron populations are evident from 8 weeks in the supragranular lamina of the motor cortex, progress to loss of CR-expressing neurons by 16 weeks, and include contrasting alteration of CR and NPY populations by end-stage disease at 20 weeks. The early involvement of both populations within the supragranular motor cortex indicates inhibitory cell deficits originate in the upper cortical layers (I-IV) of the motor cortex in the SOD1 mouse model. We found a significant increase in the length of the most distal, terminal CR-processes from 8 weeks, a number of weeks prior to their apparent loss at 16 weeks, and preceding the marked decrease in process complexity by end-stage. While we have not identified the cause of this unique regional susceptibility, it likely involves the layer II/III inhibitory and excitatory populations, and corticospinal motor neuron apical processes.

The majority of CR-interneurons reside within layer II/III, where they may directly synapse with apical processes from layer V corticospinal motor neurons and provide feedback or lateral inhibition^54^. In SOD1^G93A^ mice, it has been shown that at 8 weeks the apical dendrites of these layer V motor neurons located within layer II/III are severely degenerated and have reduced spine density, while the basal motor neuron dendrites in layer V do not exhibit overt signs of degeneration^30^. This may suggest unique regional involvement of CR-neurons is initiated by, or responding to, alterations in spine density of the apical corticospinal motor neuron dendrites within layer II/III of the motor cortex. Therefore, the dendritic inhibition of pyramidal neurons may be less effective. In support of this, the apical dendritic spines of layer V projection neurons, typically receive excitatory inputs from thalamocortical neurons and from local layer II/III neurons^55^, whereas basal spines preferentially receive inputs from distal sensory and motor nuclei^56^. Recent works suggest corticospinal motor neuron spine loss can occur at 3 weeks in the SOD1^G93A^ mouse model accompanied by increased functional excitatory synaptic activity onto layer V pyramidal neurons^28^. Hence mutant SOD1 may mediate increased excitability through early spine dynamics, leading to apical dendritic regression and inhibitory neuronal alteration; however, aberrant inhibition may also have the capacity to initiate pathology.

Close parallels can be drawn between pathology in the SOD1 mouse, and pathogenic mechanisms in the TDP-43 and HERV-K mouse models of ALS. In TDP-43^A315T^ mice there is a loss of dendritic spines on layer V projection neurons that has been shown to coincide with decreased excitability, preceding motor dysfunction and cell loss^57^,^58^,^59^. However, weeks prior to this decreased activity a population of somatostatin-expressing interneurons was found to be hyperactive, inhibiting parvalbumin interneurons that due to their direct connections with layer V neurons resulted in hyperexcitability of the layer V neurons^31^. Therefore, there is potential for early inhibitory alteration of specific cortical circuits to cause a switch in the excitable state of layer V neurons, leading to abnormal spine dynamics and a decline in neuronal function. While we find interneuron pathology from symptom onset in this study, other works using the SOD1 zebra fish have identified interneuron functional deficits as the earliest pathophysiological event. This suggests that much like the TDP-43 model, there is potential for early involvement of inhibitory microcircuits to cause a pathological switch in the excitability of projection neurons within the SOD1 motor cortex. Similarly, mice that express the human endogenous retrovirus-K in neurons also display hyperexcitability of layer V neurons, and develop a progressive motor dysfunction that includes loss of both upper and lower motor neurons with distinct reductions in the dendritic length, branch number and spine density of cortical pyramidal neurons. As the activated HERV-K virus is found specifically within neurons of sporadic ALS patients, this may suggest pathogenic mechanisms can lead to the same phenotypic neurodegenerative disease through the misregulation, or aberrant activity, of layer V neurons. While it remains unclear why interneurons within the motor cortex are vulnerable to disease, it is likely that their involvement is central to a convergence of excitatory imbalance in the disease (reviewed in^2^^,^^3^).

Cortical interneurons are essential of the regulation of normal excitability, but it is increasingly apparent that CR-interneurons in particular are susceptible to pathogenic changes in excitability. In this study the CR-expressing interneurons were reduced in number and showed morphological abnormalities. While we cannot conclude if this represents loss of the protein or loss of the cell, there were changes consistent with altered network activity. In mouse models of epilepsy and in the epileptic human hippocampus increased excitability is accompanied by the loss of CR-containing interneurons and reorganisation of their neurites^60^. This is exemplified in the sclerotic hippocampus where the density of CR-immunopositive neurons is significantly decreased, while in non-sclerotic hippocampus CR-interneurons are preserved^61^. Our finding that CR-interneurons can be found to have altered morphology as early as 8 weeks raises the possibility that they are involved in the initial alteration of the motor network in an excitable state. Although their functional impact on circuitry needs to be further investigated, it has recently been shown that the formation of CR-interneurons innervation field is an activity dependent process, with the length of axonal arbours on both multipolar and bipolar populations influenced directly by alterations in excitability^62^. Furthermore, CR-interneurons are a unique inhibitory population in the cortical circuit, as they preferentially regulate the activity of other GABAergic inhibitory populations, and subsequently the actions of principal neurons via disinhibition^63^,^64^. In this manner, not only may CR-interneurons be more susceptible to early alterations in excitability, but they also have the ability to potentiate a wide reaching inhibitory and excitatory circuit dysfunction, due to their distinctive connectivity patterns and continual alteration throughout disease. The major excitatory input to corticospinal motor neurons is a pathway from layer II/III to layer V^65^; hence interneurons located within layer II/III may disproportionately influence corticospinal motor neuron activity via disynaptic feedforward inhibition^54^. Indeed, the contrasting and somewhat unexpected involvement of NPY-interneurons may also support a continuum of pathogenic alterations triggered by altered excitability.

Neuropeptides are preferentially released during sustained neuronal stimulation^66^, and are thought to act as an endogenous neuroprotectant against increased pathogenic cortical activity^67^,^68^,^69^,^70^. Extensive literature from the epilepsy field supports a neuroprotective role for NPY as an endogenous anti-epileptic^67^. Mice lacking NPY are more susceptible to spontaneous and pharmacologically induced seizures, which can be reversed by intracerebral administration of NPY^71^,^72^. Moreover, increased synthesis and release of NPY has been reported following seizure activity^73^,^74^ and increased numbers of interneurons expressing NPY following excitotoxin treatment^75^. This indicates increased NPY expression by interneuron populations may be an advantageous intrinsic mechanism to counteract increased activity of cortical excitatory neurons. Therefore, our finding of increased density of NPY-immunoreactive neurons at end-stage may be a direct consequence of increased activity in the motor cortex.

In line with this, it is quite interesting that NPY pathology was first restricted to the upper cortical layers of the motor cortex, where CR-populations are predominately altered, but by end-stage NPY pathology was widespread throughout the entire motor cortex. This may suggest initial inhibitory deficits cause a localised change in excitability within the motor cortex, which spreads, resulting in increased NPY-immunoreactivity throughout the major excitatory pathways within the diseased motor cortex. In the context of our early results, this may suggest a late stage compensatory mechanism whereby NPY expression is upregulated to dampen the effects of altered excitability in motor circuitry. However, as we show a decreased density of NPY-interneurons at 8 weeks, excitability in the motor cortex may fluctuate throughout the disease course. In this respect, it is also interesting to note that both SOD1 and TDP-43 ALS models demonstrate the increasing involvement of neuropeptide systems, as somatostatin-interneuron numbers are reported to increase by late stages in TDP mice^31^. This is particularly important because it shows that not all interneuron populations may be involved in a similar manner in the disease, and the targeting of inhibitory populations for the restoration of normal excitability in the ALS cortex might best be considered in a subpopulation specific paradigm.

## Conclusion

Our study clearly demonstrates a novel timeline of interneuron pathology in the SOD1^G93A^ motor cortex. This pathology included the specific involvement of NPY- and CR-expressing interneuron populations. It is important to note we studied interneuron populations with immunohistochemistry; hence it remains to be determined how interneuron alterations functionally influence the disease, whether pathology is primary or secondary to excitability, and protective or pathogenic in the context of the disease. Nonetheless, we have demonstrated that changes originate in the upper cortical layers of the motor cortex from early symptom onset, and progress to involve the entire motor cortex by end-stage disease. While the role of cortical components in motor neuron circuitry is only just beginning to be elucidated, it is apparent that specific inhibitory populations may have an underappreciated role in the disease. It will be important for future electrophysiological studies to determine if this continuum of interneuron alteration might compromise the cortical motor network and will be essential for developing effective therapeutics aimed at the restoration of normal excitability for the treatment and prevention of ALS.

## Methods

Male transgenic mice carrying a high copy number of the human SOD1^G93A^ mutation [strain 004435 B6.Cg-Tg(SOD1^G93A^)1Gur.J - backcrossed for more than 10 generations on a C57BL/6 background] [Jackson Laboratory (CA, USA) (http://www.jax.org/strain/004435)], and their wild-type littermates were used for histological analyses. Mice were genotyped and copy number determined (25 ± 2) using a multiplexed quantitative polymerase chain reaction according to the Jackson Laboratory protocol: http://www.jax.org. Animals were housed in individually ventilated cages at 20 ^o^C, on a 12 hour light-dark cycle, with access to food and water *ad libitum*. Presence of the SOD1^G93A^ transgene was assessed according to standard protocols^76^. All procedures were approved by the Animal Ethics Committee of the University of Tasmania and conducted in accordance with the Australian Code of Practice for the Care and Use of Animals for Scientific Purposes, 2013.

### Preparation of time-series cortical tissue from SOD1^G93A^ and WT mice

*SOD1*^*G93A*^ transgenic mice and age-matched wild-type controls were sacrificed from symptom onset in the *SOD1*^*G93A*^ mouse^27^ and assessed at defined time points until end-stage in the disease course: 8 weeks (symptom-onset), 12 weeks, 16 weeks and 20 weeks of age (end-stage). The final time point was determined as an ethical stage preceding the 157-day typical life span in this *SOD1*^*G93A*^ mouse model^26^, and the earliest time point investigated when distal pathology has been previously described in our laboratory^77^. Mice were terminally anaesthetised (sodium pentobarbitone, 140 mg/kg, i.p) and transcardially perfused with 4% paraformaldehyde (PFA; w/v) [(in 0.01 M phosphate buffered saline (PBS)]. For each of the above time points, the cortex was obtained from six animals per genotype per time point. The brains were post-fixed in 4% PFA overnight at 4 ^o^C, then stored at 4 ^o^C in 0.01 M PBS containing 0.1% w/v sodium azide (Sigma Aldrich, Australia).

### Tissue processing

The brain was cut at Bregma −4.00 mm, and the anterior portion cryoprotected with increasing concentrations of sucrose (4%, 16%, 30%) dissolved in 0.01 M PBS. Serial coronal cryostat sections (40μm) were generated using a Leica CM 1850 cryostat (Biosystems, Australia) and collected as free-floating sections into 24 well plates (Corning Life Sciences, USA) containing sodium azide, kept in sequential order and stored at 4 ^o^C until processed for immunohistochemistry.

### Immunolabeling for interneuron markers in the SOD1^G93A^ and WT cortex

For analysis of cortical interneuron pathology, free-floating sections were processed using standard immunohistochemical methods^57^,^78^. Cortical interneurons were identified by the expression of calcium binding proteins: calbindin (CB), calretinin (CR) and parvalbumin (PV), or by neuropeptides: neuropeptide Y (NPY), vasoactive intestinal peptide (VIP) and somatostatin (SOM)^19^. Every tenth serial sections (400 μm apart) was incubated with antibodies recognizing cell-type specific interneuron markers diluted in 0.01 M PBS containing 0.3% Triton-X-100 (see Table 1 for antibody dilutions). Following washes (3 × 0.01 M PBS, 10 min) to remove excess unbound antibodies, sections were incubated with alexa-fluor conjugated secondary antibodies (1:1000, Thermo-Fisher Scientific, Australia) diluted in 0.01 M PBS at room temperature for 2hrs, followed by DAPI staining (4′,6-diamidino-2-phenylindole, 1/50000, Thermo-Fisher Scientific, Australia). After further washes (3 × 0.01 M PBS, 10 min), sections were mounted onto glass slides and coverslipped using fluorescent PermaFluor™ aqueous mounting medium (Thermo-Fisher Scientific Australia Pty Ltd, Australia). Specificity of all antibodies was verified by incubating sections with the corresponding secondary antibody without pre-incubation of primary antibody.

### Confocal microscopy and cell counting

Immunofluorescence was captured using a Zeiss LSM 510 DuoScan confocal microscope (Carl Zeiss Microscopy, Germany), running Zen software (V3.2, 2008) equipped with Ar488 and HeNe543 lasers. Cell bodies were quantified blind to genotype in the supragranular (layers I-IV) and infragranular lamina (layers V-VI) of the primary motor and secondary somatosensory cortices, comparable coronal sections were selected from 1.18mm to −0.58mm relative to bregma (Sections 21–36 according to the Paxinos and Franklin Mouse Brain Atlas^79^ (Fig. 1). A plan-apochromat 20x objective (N.A. 0.8, Zeiss) was used to generate z-plane images with 2μm intervals through 16 μm of tissue depth. Primary motor and secondary somatosensory cortices were identified by anatomical landmarks referring to the appearance of the lateral ventricles, the shape of the third ventricle and the appearance of the anterior commissure and corpus callosum, as visualized with DAPI staining and according to the Allen Mouse Brain Atlas (© Allen Institute for Brain Science: http://mouse.brain-map.org) (Fig. 1). Immunopositive neurons (cells with positive labelling in cell soma) were counted using Image J software (National Institutes of Health, USA) with the integrated Cell Counter plugin utilising Nissl staining to identify cortical layers. To compare densities of immunopositive neurons in SOD1^G93A^ and wild-type mice, all neurons within the regions of interest (ROI) were manually marked, counted and the density calculated using the area of the ROI (values are given in cells/mm^2^). The densities were then averaged across animals with 4 sections per cortical region per mouse included in analyses.

### Morphological analyses

For analysis of neurite labelling patterns, 40 μm coronal tissue sections were used to generate Z-stack images of neurons with 1 μm intervals through 16 μm of tissue depth within the motor cortex. Neurons with full arbours within z-stack were analysed using the cell tracing software Neurolucida^TM^ (MBF Bioscience, USA) with the z-stack margins set to include one complete cell layer. For quantification of neurites immunoreactive for calretinin (CR), neurons were traced through stacks with processes marked, and images then exported to Neurolucida^TM^ Explorer II (MBF Bioscience, USA). Branched structure analysis was used to analyse the number, area and length of primary, secondary, tertiary and quaternary order neurite processes, encompassing both axons and dendrites, of CR-labelled neurons.

### Statistical analyses

Neuronal density was analysed using a two-way analysis of variance followed by Bonferroni post hoc tests (GraphPad Prism, Version 6.0) for group and regional comparisons. Overall group differences (main effects of genotype) were identified using non-parametric two-tailed *t*-tests. To assess neuronal densities across the disease course, three-way analysis of variance was used for comparisons of group and cortical regions between different time points (SPSS, Version 20). All variables were tested for statistical interaction, with any significant interactions included in the model. Statistical significance was set at *P* < 0.05. Average values were expressed as means ± standard error of mean.

## Additional Information

**How to cite this article:** Clark, R. M. *et al*. Calretinin and Neuropeptide Y interneurons are differentially altered in the motor cortex of the SOD1^G93A^ mouse model of ALS. *Sci. Rep.* **7**, 44461; doi: 10.1038/srep44461 (2017).

**Publisher's note:** Springer Nature remains neutral with regard to jurisdictional claims in published maps and institutional affiliations.

## Figures and Tables

**Table 1:** Primary Antibodies.

Antigen	Description of Immunogen	Source, Host Species, Cat#, Clone or Lot#, RRID	Concentration Used
Calbindin D-28k	Calcium-binding protein of the EF-hand family related to calmodulin and troponin-C	Millipore, rabbit polyclonal, Cat#AB1778 Lot# RRID:AB_2068336	1:1000 μl (IHC)
Calretinin	Calcium-binding protein of the EF-hand family related to calbindin D-28k and calmodulin	Swant, mouse monoclonal, Cat#6B3 Lot#010399 RRID:AB_1000330	1:1000 μl (IHC)
Parvalbumin	Calcium-binding protein of the EF-hand family related to calmodulin and troponin-C	Swant, mouse monoclonal, Cat#235 Lot#10-11 (F) RRID:AB_10000343	1:1000 μl (IHC)
Neuropeptide Y	Neuropeptide Y conjugated to bovine thyroglobulin (BTg) with glutaraldehyde	Immunostar, rabbit polycloncal, Cat#22940 Lot#1112001 RRID:AB_572253	1:500 μl (IHC)
Somatostatin	Synthetic peptide coupled to keyhole limpet hemocyanin (KLH) with carbodiimide (CDI) linker	Immunostar, rabbit polyclonal, Cat#20067 Lot#216002 RRID:AB_572264	1:1000 μl (IHC)
Vasoactive Intestinal Peptide	Porcine VIP coupled to bovine thyroglobulin (BTg) with carbodiimide (CDI) linker	Immunostar, rabbit polyclonal, Cat#20077 Lot#1339001 RRID:AB_572270	1:1000 μl (IHC)
